# HLA and pathogens in myalgic encephalomyelitis/chronic fatigue syndrome (ME/CFS) and other post-infection conditions

**DOI:** 10.1038/s41598-025-21230-z

**Published:** 2025-10-24

**Authors:** Apostolos P. Georgopoulos, Lisa M. James, Philip K. Peterson

**Affiliations:** 1 The HLA and Chronic Diseases Research Groups, Brain Sciences Center, Department of Veterans Affairs Health Care System, Minneapolis VAMC, One Veterans Drive, Minneapolis, MN 55417 USA; 2 Department of Neuroscience, University of Minnesota Medical School, Minneapolis, MN USA; 3 Institute for Health Informatics, University of Minnesota Medical School, Minneapolis, MN USA; 4 Department of Psychiatry, University of Minnesota Medical School, Minneapolis, MN USA; 5 Department of Medicine, University of Minnesota Medical School, Minneapolis, MN USA

**Keywords:** Myalgic Encephalomyelitis/Chronic Fatigue Syndrome (ME/CFS), Human herpes viruses, Human Leukocyte Antigen (HLA), Binding affinity, Immunogenetics, Adaptive immunity, Viral infection

## Abstract

Viral infections have been widely implicated in Myalgic Encephalomyelitis/Chronic Fatigue Syndrome (ME/CFS) pathogenesis. Recent evidence has also identified certain Human Leukocyte Antigen (HLA) alleles that are significantly associated with ME/CFS risk/protection. Here we tested the hypothesis that ME/CFS risk or protection conferred from those HLA alleles is associated with binding affinity to antigens of HHV viruses, a critical step in initiating the adaptive immune system response to foreign antigens. Specifically, we determined in silico the predicted binding affinity of two susceptibility alleles (C*07:04, DQB1*03:03) and two protective alleles (B*08:01, DPB1*02:01) to > 10,000 antigens of the 9 Human Herpes Viruses (HHV1, HHV2, HHV3, HHV4, HHV5, HHV6A, HHV6B, HHV7, HHV8) which have been implicated in the etiology of ME/CFS. We found that the binding affinity of all HHV antigens to the susceptibility alleles was significantly weaker than the binding affinity to the protective alleles (*P* < 0.001). In fact, none of the HHV antigens showed strong binding to the susceptibility alleles, in contrast to the strong bindings showed by the protective alleles. These findings are in keeping with the hypothesis that the effect of a putative HHV insult in contributing to ME/CFS is modulated by the host’s HLA immunogenetic makeup. We speculate that strong HLA-antigen binding likely protects against ME/CFS via elimination of virus antigens; conversely, weak HLA-antigen binding may permit persistence of foreign antigens, contributing to ME/CFS and other chronic conditions. Finally, with respect to the latter, we determined the binding affinities to the 4 HLA alleles above to pathogens causing two chronic diseases with very similar symptomatology to ME/CFS, namely Long COVID and post-treatment Lyme disease syndrome (PTLDS). We found that the 2 ME/CFS susceptibility HLA alleles above had very weak binding with SARS-CoV-2 virus glycoprotein (involved in Long COVID) and 5 proteins of *Borrelia burgdorferi* (involved in PTLDS), in contrast to the ME/CFS protective alleles that showed strong bindings. These findings support the hypothesis that ME/CFS, long COVID and PTLDS are caused by persistent pathogenic antigens that could not be eliminated due to inadequate protection by the patient’s HLA makeup.

**Supplementary Information:**

The online version contains supplementary material available at 10.1038/s41598-025-21230-z.

## Introduction

### Myalgic encephalomyelitis/chronic fatigue syndrome (ME/CFS): clinical

ME/CFS is a chronic, debilitating condition that affects approximately 1% of the population^1^, probably an underestimate^2^. Although case definitions vary^3,4^, symptoms of ME/CFS include impairing fatigue, post-exertional malaise, cognitive dysfunction, and joint pain, among others. Dysregulation of multiple physiological processes has been reported, affecting immune, inflammation, metabolic, and mitochondrial function, among others^5^. Moreover, the presence of widespread neuroinflammation in cortical areas and subcortical nuclei has been documented using positron emission tomography^6^. Overall, ME/CFS has major impacts on quality of life, including significant social and occupational impairment and disability^3,7^. A comprehensive and detailed investigation and assessment of multiple clinical, behavioral, immunological and various biomarker aspects of post-infectious ME-CFS has been published recently^8^.

### ME/CFS: etiology

The etiology of ME/CFS is uncertain; viral infections, including several human herpes viruses, have been prominently implicated as a potential trigger^9,10^, and there is evidence of genetic predisposition to ME/CFS^11,12^. In 2022, we^13^ proposed that ME/CFS may be, at least in part, due to persistent viral antigens (i.e., fragments of viral proteins) resulting from low binding affinity of those antigens to the Human Leukocyte Antigen (HLA) molecules carried by the patient. The HLA region, which is located on chromosome 6, is the most highly polymorphic region of the human genome^14^ and is aimed at host protection against viruses and other foreign antigens. Each individual carries 12 HLA alleles—two of each of the classic HLA Class I (HLA-I) genes (HLA-A, HLA-B, and HLA-C) and two of each of the HLA Class II (HLA-II) genes (HLA-DPB1, HLA-DQB1, HLA-DRB1)—that determine the repertoire of foreign antigens that can bind with sufficient affinity to HLA molecules to mount an immune response, namely killing the infected cell and production of suitable antibodies. Here, we formally test the hypothesized role of HLA-antigen binding with putative viral antigens in ME/CFS risk and protection.

### ME/CFS: HLA

In a recent thorough and well-powered study^12^ of HLA and ME/CFS, two HLA alleles were identified that occurred in significantly higher frequency in the ME/CFS group (N = 426) than the control group (N = 4511 healthy and ethnically matched participants), namely C*07:04 and DQB1*03:03. In addition, 2 alleles with independent, significantly higher frequencies in the control group, presumably protective against ME/CFS were identified: B*08:01 and DPB1*02:01. Based on our proposal that ME/CFS is influenced by persistent viral antigens due to HLA-antigen mismatch^13^, we hypothesized that the latter alleles were effective in facilitating elimination of pathogenic viral persistent antigens, thus rendering exposed individuals less susceptible to ME/CFS whereas, in contrast, the risk alleles were hypothesized to have low binding affinity to virus antigens, hindering effective immune response to facilitate their elimination, thus allowing their persistence. Here we tested that hypothesis by determining in silico the predicted best binding affinity (PBBA) of the 4 alleles above to antigens of the 9 known Human Herpes Viruses (HHV), all of which have been implicated in ME/CFS^10^, with the expectation that the HLA molecules of the risk alleles (C*07:04, DQB1*03:03) would bind weakly to the viral antigens, in contrast to those of the protective alleles (B*08:01, DPB1*02:01) which would bind strongly.

### Long COVID

The resemblance in symptomatology between Long COVID and ME/CFS (fatigue, neurocognitive problems, postexertional tiredness, etc.) was noted early on^13–15^. In 2022 we hypothesized that Long COVID might be due to persistent pathogenic SARS-CoV-2 fragments^13^, a hypothesis that has since been supported by the identification of SARS-CoV-2 RNA and viral fragments in long COVID^16,17^. In that paper^13^ we hypothesized that the persistence of pathogenic antigens is due to the inability of the patient’s HLA to effectively bind and present these antigen fragments, and proposed this as a common mechanism underlying Long COVID, ME/CFS, and Gulf War Illness, a disorder with symptomatology highly similar to ME/CFS, that is linked to anthrax vaccine administration during the 1990–91 Gulf War^18^. The HLA-antigen persistence hypothesis was proposed in 2018^19^. In this study we tested the hypothesis that the same HLA alleles that were identified as risk or protective for ME/CFS^12^ might also share this property for Long COVID by determining exhaustively their binding affinities to peptides of the SARS-CoV-2 spike glycoprotein.

### Post treatment lyme disease syndrome (PTLDS)

Finally, here we applied the same rationale for another syndrome with symptomatology overlapping those of ME/CFS and Long COVID, namely the PTLDS. Lyme disease is a tick-born disease caused by the spirochete *Borrelia burgdorferi* with very similar symptomatology to ME/CFS^20^*.* In the large majority of cases, if treated early, recovery is complete. However, in a small fraction of patients, “The constellation of symptoms such as fatigue, cognitive dysfunction, and musculoskeletal pain that persist beyond 6 months and are associated with disability have been termed post-treatment Lyme disease syndrome (PTLDS)”^21^. A persistent antigen from *B. burgdorferi* associated with persistent post-Lyme arthritis is peptidoglycan, a major component of the *B. burgdorferi* envelope^22^. It is not known whether, or to what extent, additional antigens may exist and be involved in PTLDS, affecting the brain and other organs, besides the joints. In this study we tested the hypothesis that the ME/CFS-related HLA alleles identified by Lande et al.^12^ might also share this property for PTLDS by determining exhaustively their binding affinities to peptides of 5 *B. burgdorferi* proteins.

## Results

### HHV

**Fig. 1:**
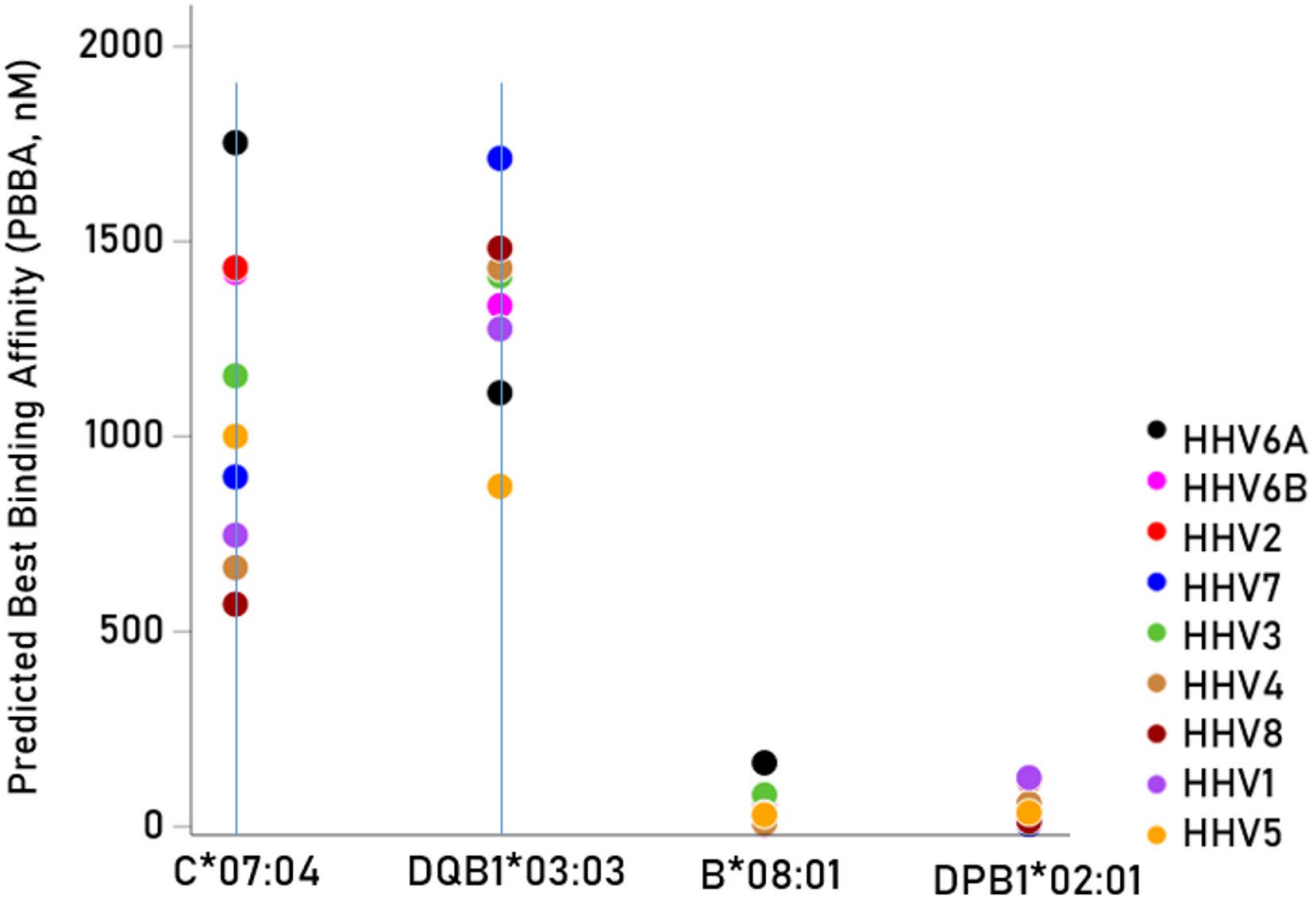
Predicted Best Binding Affinities (PBBA) for the 4 HLA alleles and 9 HHV tested. nM, nanomolar. Lower values indicate better (stronger) binding.

**Fig. 2:**
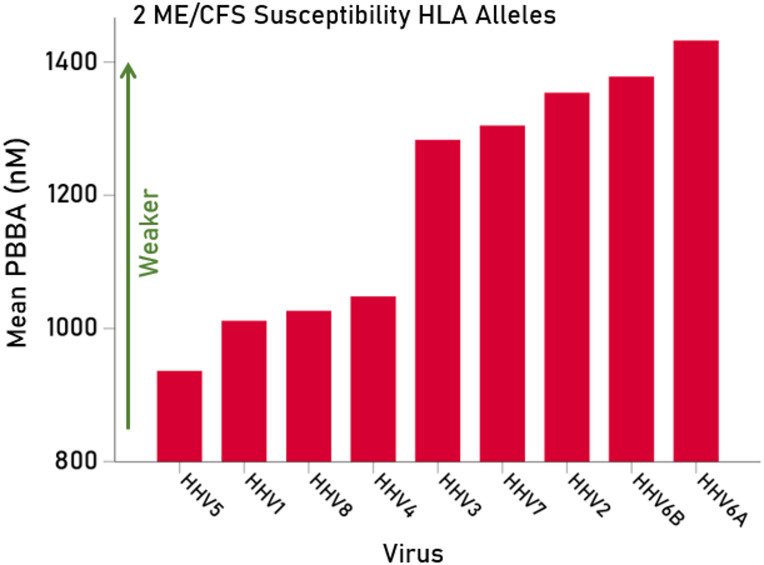
The average PBBAs of the 2 risk alleles (C*07:04, DQB1*03:03) for each HHV tested are plotted ranked from low to high PBBA; higher PBBAs indicated weaker binding.

The 9 HHV antigens tested are shown in Table 1 together with details of the protein ID and the number of peptides tested. A grand total of 10,528 viral protein peptides of these viral proteins were tested against the 4 ME/CFS related HLA alleles. Table 2 shows the predicted best binding affinities (PBBA) of the 2 risk and 2 protective alleles^12^ to the antigens tested. It can be seen that none of the HHV antigens were associated with strong binding affinity to the ME/CFS risk alleles; in contrast, the majority of HHV antigens were associated with strong binding affinity to the ME/CFS protective alleles. Descriptive summary statistics are given in Table 3. The PBBAs for the protective alleles were significantly stronger (i.e. with lower IC_50_ values) than those of the risk alleles (*P* < 0.001, Mann–Whitney test) (Fig. 1). The average PBBAs per viral antigen tested for the ME/CFS risk alleles are shown in the bar graph of Fig. 2. It can be seen that all 9 HHVs were characterized by weak binding affinity; HHV6A had the highest PBBA (i.e. weakest binding affinity). Finally, we evaluated the association between the log-transformed odds ratio of ME/CFS^12^ and the log-transformed mean PBBA of the 9 HHVs (Table 4) and found a strong correspondence (Pearson $r$ = 0.956, *P* = 0.044, N = 4). That is, alleles associated with lowest (i.e., stronger) binding to virus antigens are associated with reduced odds (risk) of ME/CFS.

**Table 1:** Viral antigens used.

	Pathogen	Protein	UniprotKB ID	AA	HLA-I	HLA-II
ME/CFS (HHV)						
1	HHV1	Envelope glycoprotein D	Q69091	394	386	380
2	HHV2	Envelope glycoprotein D	P03172	393	385	379
3	HHV3	Envelope glycoprotein E	Q9J3M8	623	615	609
4	HHV4	Envelope glycoprotein B	P03188	897	889	883
5	HHV5	Envelope glycoprotein B	P06473	906	898	892
6	HHV6A	Envelope glycoprotein Q2	P0DOE0	214	206	200
7	HHV6B	Envelope glycoprotein Q1	Q9QJ11	516	508	502
8	HHV7	Envelope glycoprotein H	P52353	690	682	676
9	HHV8	Envelope glycoprotein H	F5HAK9	730	722	716
Total					5291	5237
Total peptides tested					10,528	
Total tested (× 4 HLA alleles)					42,112	
Long COVID (SARS-CoV-2)						
	SARS-CoV-2	Spike glycoprotein	P0DTC2	1273	1265	1259
Total peptides tested					2524	
Total tested (× 4 HLA alleles)					10,096	
PTLDS (*B. burgdorferi*)						
1	*B. burgdorferi*	Outer surface protein A	P0CL66	273	265	259
2	*B. burgdorferi*	Outer surface protein C	Q07337	210	202	196
3	*B. burgdorferi*	Decorin-binding protein A	O50917	191	183	177
4	*B. burgdorferi*	OppA-2	Q6RH12	107	99	93
5	*B. burgdorferi*	Variable large protein	O06878	356	348	342
Total					1097	1067
Total peptides tested					2164	
Total tested (× 4 HLA alleles)					8656	
Grand total					60,864	

The last two columns are the numbers of 9-mers (HLA-I) and 15-mers (HLA-II) analyzed.

**Table 2:** PBBA (nM) of the 2 ME/CFS risk and 2 protective alleles^12^ for the 9 viral antigens tested (Table 1).

	Virus	Risk				Protective			
		C*07:04		DQB1*03:03		B*08:01		DPB1*02:01	
		PBBA (nM)	S	PBBA (nM)	S	PBBA (nM)	S	PBBA (nM)	S
1	HHV1	747.6	0	1275.7	0	32.1	1	125.8	0
2	HHV2	1432.7	0	1275.7	0	45.5	1	117.8	0
3	HHV3	1155.4	0	1411.2	0	81.8	0	42.3	1
4	HHV4	664.6	0	1431.9	0	9.2	1	59.3	0
5	HHV5	1000.1	0	872.9	0	30.3	1	36.3	1
6	HHV6A	1753.1	0	1111.7	0	163.8	0	45	1
7	HHV6B	1421.1	0	1335.5	0	4.8	1	22.5	1
8	HHV7	897	0	1712.9	0	24.2	1	5.6	1
9	HHV8	570.5	0	1482.7	0	33.8	1	14.3	1

S = 0/1 indicates weak (PBBA > 50 nM) or strong (PBBA < 50 nM) binding, respectively. nM, nanomolar.

**Table 3:** Descriptive statistics of PBBAs of the 4 alleles and 9 HHV tested.

	Risk		Protective	
	C*07:04	DQB1*03:03	B*08:01	DPB1*02:01
Mean	1179.1 nM	1333.7 nM	65.3 nM	42.7 nM
SD	617.7	259.8	84.7	38.3
SEM	171.3	72	23.5	10.6
Minimum	570.5	872.9	4.8	5.6
Maximum	2862.6	1863.4	310.6	125.8
Median	1000.1	1275.7	32.1	35.5
IQR	720.8	306	55.2	38.7
25th Percentile	706.1	1151.3	19.8	13.4
75th Percentile	1426.9	1457.3	75	52.1
Odds Ratio (OR)^7^	2.03	1.46	0.7	0.7

SD, standard deviation; SEM, standard error of the mean. nM, nanomolar. See text for details.

**Table 4:** Natural log-transformed values of odds ratio^12^ and mean PBBA (Table 3) for the 4 HLA alleles and 9 HHV tested. nM, nanomolar.

Allele	ln(Mean PBBA)	ln(OR)
C*07:04	6.977 nM	0.7419
DQB1*03:03	7.188	0.4055
B*08:01	3.856	− 0.3567
DPB1*02:01	3.953	− 0.3567

### SARS-CoV-2

We evaluated the PBBA of the SARS-CoV-2 antigen for the ME/CFS risk/protective alleles by testing exhaustively 2524 peptides (Table 1). As shown in Table 5, for HLA-I, the PBBA for the ME/CFS risk allele C*07:04 (362.2 nM) was 18.2 × weaker than the protective allele B*08:01 (19.9 nM). Similarly, for HLA-II, the PBBA for the ME/CFS risk allele DQB1*03:03 (614.1 nM) was 114.1 × weaker than the protective allele DPB1*02:01 (5.38 nM).

**Table 5:** PBBAs of the 4 alleles with SARS-CoV-2 and *B. burgdorferi* antigens. nM, nanomolar.

	Allele	SARS-CoV-2	*B. burgdorferi*
Risk	C*07:04	362.2 nM	1410.7 nM
	DQB1*03:03	614.1	1028.2
Protective	B*08:01	19.9	68.3
	DPB1*02:01	5.38	71.4

### *B. burgdorferi*

Here we evaluated the PBBA of five *B. burgdorferi* antigens with the ME/CFS alleles by testing exhaustively 2164 peptides (Table 1). For HLA-I, the PBBA (across the 5 antigens) for the ME/CFS risk allele C*7:04 (1410.7 nM) was 20.7 × weaker than the protective B*08:01 allele (68.3 nM) (Table 5). For HLA-II, the PBBA for the ME/CFS risk allele DQB1*03:03 (1028.2 nM) was 14.4 × weaker than the protective allele DPB1*02:01 (71.4 nM).

## Discussion

A recent study identified HLA alleles that are associated with ME/CFS risk or protection^12^. Here we tested the hypothesis that risk or protection conferred by those alleles would be associated to the strength in binding affinity to common viruses. As expected, the predicted binding affinities of previously identified ME/CFS HLA risk alleles (C*07:04, DQB1*03:03) to virus antigens are significantly weaker than the predicted binding affinities of protective HLA alleles (B*08:01, DPB1*02:01). Furthermore, we exhaustively tested the predicted binding affinity of 10,528 amino acid sequences (Table 1) of 9 HHV antigens to the HLA alleles implicated in ME/CFS risk and found that none of them met the threshold for strong binding affinity (IC_50_ < 50 nM)^23^. On average, the risk alleles were characterized by weak binding affinity to the viruses investigated; specifically, the predicted best binding affinity was weak for 100% of the HHVs with the risk alleles. In contrast, strong predicted best binding affinity was documented for 78% (7 of 9) of HHVs with B*08:01 and 67% (6 of 9) with DPB1*02:01, the two protective alleles. Notably, the mean binding affinity of the alleles with the 9 HHVs was significantly associated with the odds ratio of ME/CFS documented by previous researchers^12^. HHVs are nearly ubiquitous neurotropic viruses that establish latency after initial infection and are intermittently reactivated in contexts such as stress or immunosuppression^24^. With regard to ME/CFS, a recent meta-analysis documented that of the HHVs, the highest odds of ME/CFS were associated with HHV6 and HHV7 and particularly with their co-infection^10^. Similarly, here, HHV6a, HHV6b, and HHV7 were among those with the weakest binding affinities to the risk alleles, further supporting their influence on ME/CFS. Remarkably, SARS-CoV-2 and *Borrelia burgdorferi* antigens were similarly characterized by weak affinity to the ME/CFS risk alleles and substantially stronger affinity to the ME/CFS protective alleles. Taken together, these findings suggest that risk conferred by certain HLA alleles is likely attributed to limited binding with antigens of virus or bacterial pathogens, hindering the immune system response aimed at targeting the offending pathogen. We have hypothesized that absence of sufficient HLA-antigen binding permits foreign antigens to persist, contributing to chronic symptoms characteristic of ME/CFS and other chronic conditions such as long-COVID and Lyme disease^13^.

HLA molecules, which are cell-surface glycoproteins encoded by genes on the short arm of chromosome 6, play a critical role in adaptive immunity via presentation of foreign antigens to T lymphocytes to stimulate immune defense^25^. Both HLA Class I and Class II bind and present foreign antigen peptides to T cells, albeit via different mechanisms and with different outcomes. HLA Class I molecules, which are expressed on all nucleated cells, present endogenous antigens including virus-induced proteins to cytotoxic CD8 + T cells, signaling destruction of infected cells. HLA Class II molecules, which are restricted to antigen presenting cells, present endocytosed exogenous antigens to T cell receptors of CD4 + lymphocytes, stimulating antibody production and long-term immunity. The two main HLA classes work together to quickly eliminate pathogens and protect against future infection, assuming sufficient binding between HLA and a given peptide. HLA, however, is extremely polymorphic^26^, contributing to variations in the binding groove that impact binding affinity^27^ and disease risk^28^. Lande and colleagues^12^ previously documented HLA Class I and Class II alleles that were associated with risk to (and protection against) ME/CFS. The present finding that neither the Class I (C*07:04) nor Class II (DQB1*03:03) risk alleles have strong binding affinity to any of the common pathogens we evaluated points to limited effectiveness of both arms of adaptive immunity. This stands in stark contrast to the strong binding affinity of the protective alleles (B*08:01 and DPB1*02:01) with the same pathogens, likely promoting robust adaptive immune responses to eliminate and/or neutralize pathogens, thereby protecting against ME/CFS, long-COVID, and Lyme disease.

Although the present analyses focused primarily on HLA-HHV antigen binding affinity with regard to ME/CFS, the findings extend to other conditions, particularly chronic multisymptom illnesses that are characterized by similar symptoms as ME/CFS, such as long-COVID^29–31^ and PDLS^21^. Several previous studies have documented the influence of HLA on SARS-CoV-2 infection and on Lyme disease^32–36^; limited research has evaluated HLA with regard to Long-COVID [37; c.f., 38]. Binding of HLA with peptides from viral and bacterial antigens is a key initial step in initiating adaptive immunity against pathogens to facilitate their elimination. Without sufficient binding affinity, the pathogens may persist. Indeed, fragments of SARS-CoV-2 and *Borrelia burgdorferi* protein have been identified in patients suffering from Long-COVID or Lyme disease^16,17,22^. Based on the present findings, we would expect that persistence to be more likely in the alleles that were predicted to bind weakly (C*07:04; DQB1*03:03) with all of the pathogens investigated. Of note, previous research has shown that HLA-C*07:04 is among the weakest binders to the SARS‐Cov‐2 protein^39^. The finding that the same alleles that confer risk to ME/CFS^12^ are associated with weak affinity not only for HHVs implicated in ME/CFS, but also for SARS-CoV-2 and *Borrelia burgdorferi* proteins suggests that insufficient HLA-antigen binding is a possible common thread contributing to a range of chronic multisymptom illnesses. Indeed, the overlapping symptoms of ME/CFS, long-COVID, and PTLDS—namely, fatigue, pain, cognitive dysfunction, postexertional malaise—may reflect a common set of symptoms stemming from various infectious insults similar to the common symptoms of fever and malaise accompanying acute infections by diverse pathogens (viruses, bacteria, etc.).

Despite the broader implications of this study’s novel findings, several limitations and future directions are worth noting. First, the analyses here were limited to 4 HLA alleles that were previously documented to be associated with ME/CFS risk or protection^12^. Given the extreme polymorphism^12^ and geographic variability^40,41^ of HLA, it possible that other HLA alleles not investigated here may also contribute to ME/CFS risk/protection based on their binding affinity with foreign pathogens. Similarly, it is certainly likely that weak HLA binding to pathogens beyond the HHVs studied here (e.g., enteroviruses, parvoviruses)^9,10^ may be involved in ME/CFS. Finally, the present analyses focused on the 4 ME/CFS alleles do not preclude influence of other HLA alleles on Long-COVID and PTLDS.

## Materials and methods

### Antigens

We tested a total of 15 protein antigens of pathogens (Table 1), including 9 antigens of human herpes viruses (HHV), the spike glycoprotein of SARS-CoV-2 virus, and 5 antigens of Borrelia burgdorferi (*B. burgdorferi*). The amino acid (AA) sequences of those proteins were obtained from the Uniprot website (https://www.uniprot.org/uniprotkb/)^42^ and are shown in Table S1 (Supplementary Material).

### HLA alleles

We investigated 2 ME/CFS risk HLA alleles (C*07:04, DQB1*03:03) and 2 protective alleles (B*08:01, DPB1:02:01) reported by Lande et al.^12^.

### In silico determination of predicted best binding affinities (PBBA) to pathogen antigens

**Fig. 3:**
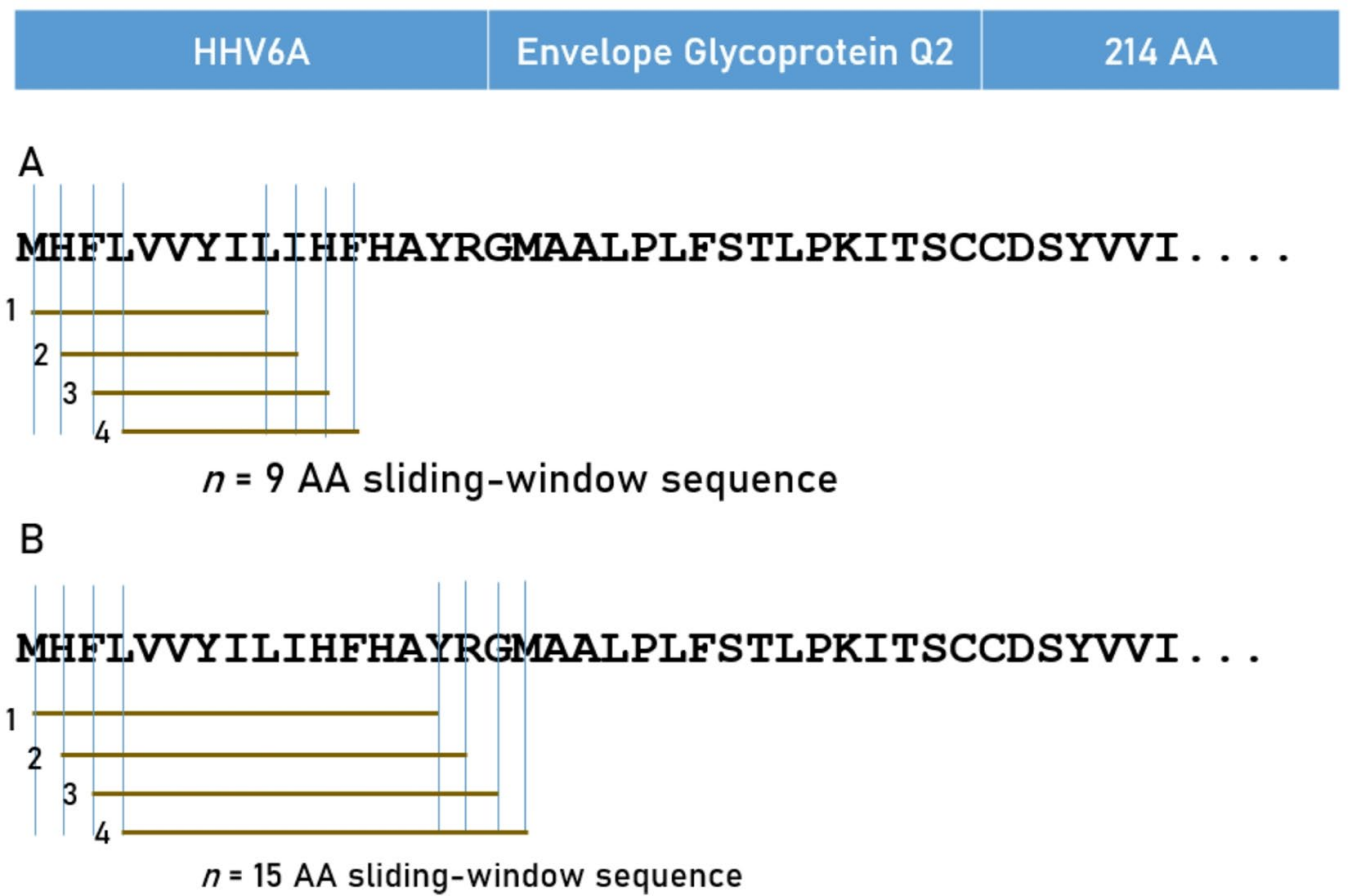
Schematic diagram to illustrate the sliding window approach to estimate in silico binding affinities of HLA alleles to one viral antigen (HHV6A).

Predicted binding affinities were obtained for antigen peptides using the Immune Epitope Database (IEDB) NetMHCpan (ver. 4.1) tool^43,44^. More specifically, we used the sliding window approach^45–47^ to test exhaustively all possible linear 9-mer (for HLA-I predictions) and 15-mer (for HLA-II predictions) peptides of the 15 antigens analyzed (Table 1). The method is illustrated in Fig. 3. For each pair of peptide-HLA molecule (pHLA-I and pHLA-II) tested, this tool gives, as an output, the IC_50_ of the predicted binding affinity; *the smaller the IC*_*50*_*, the stronger the binding affinity*. An IC_50_ value of < 50 nM (nanomolar) is regarded strong^23^. The predicted best binding affinity (PBBA) for each HLA-antigen pair was the minimum IC_50_ value of all peptides tested in the pair. Given a protein of *N* amino acid length and an peptide length of *k* AA, there are *N-k* binding affinity predictions, i.e. *N-k* + 1 IC_50_ values returned by the prediction tool. The numbers of peptides tested for each antigen and HLA Class (HLA-I, HLA-II) are given in Table 1.

### Statistical analyses

The IBM-SPSS statistical package (version 30.0.0.0 172) was used for implementing statistical analyses. Standard statistical methods were used; all P-values reported are 2-sided, $a=0.05$.

## Supplementary Information

Below is the link to the electronic supplementary material.

Supplementary Material 1

## Data Availability

All information was obtained from freely accessible websites and, as such, is publicly and freely available. The antigen protein sequences obtained from Uniprot [ref.^42^: https://www.uniprot.org/uniprotkb] are provided in Table S1. The binding affinities of each 9-mer (HLA-I) and 15-mer (HLA-II) pathogen protein sequence to HLA was exhaustively tested using IEDB [ref.^43^: http://tools.iedb.org/mhci/]. The n-mer peptide sequence with the predicted best binding affinity for each HLA antigen is provided in Table S2.
