# Supplementary material for: HLA and pathogens in myalgic encephalomyelitis/chronic fatigue syndrome (ME/CFS) and other post-infection conditions
Source: Sci Rep. 2025 Oct 24;15:37303. doi: 10.1038/s41598-025-21230-z (PMC12552462; doi:10.1038/s41598-025-21230-z)
Supplement: Supplementary file 1 — Supplementary Material 1 [file 41598_2025_21230_MOESM1_ESM.docx]

**Table S1.** Amino acid sequences of the 15 virus and bacterial proteins investigated (Table 1). Protein labels are from Uniprot (https://www.uniprot.org/uniprotkb/)

| HHV1: Q69091 · GD_HHV11 | Envelope glycoprotein D | 394 AA |
| --- | --- | --- |

MGGAAARLGAVILFVVIVGLHGVRSKYALVDASLKMADPNRFRGKDLPVLDQLTDPPGVRRVYHIQAGLPDPFQPPSLPITVYYAVLERACRSVLLNAPSEAPQIVRGASEDVRKQPYNLTIAWFRMGGNCAIPITVMEYTECSYNKSLGACPIRTQPRWNYYDSFSAVSEDNLGFLMHAPAFETAGTYLRLVKINDWTEITQFILEHRAKGSCKYALPLRIPPSACLSPQAYQQGVTVDSIGMLPRFIPENQRTVAVYSLKIAGWHGPKAPYTSTLLPPELSETPNATQPELAPEDPEDSALLEDPVGTVAPQIPPNWHIPSIQDAATPYHPPATPNNMGLIAGAVGGSLLAALVICGIVYWMRRHTQKAPKRIRLPHIREDDQPSSHQPLFY

| HHV2: P03172 · GD_HHV23 | Envelope glycoprotein D | 393 AA |
| --- | --- | --- |

MGRLTSGVGTAALLVVAVGLRVVCAKYALADPSLKMADPNRFRGKNLPVLDRLTDPPGVKRVYHIQPSLEDPFQPPSIPITVYYAVLERACRSVLLHAPSEAPQIVRGASDEARKHTYNLTIAWYRMGDNCAIPITVMEYTECPYNKSLGVCPIRTQPRWSYYDSFSAVSEDNLGFLMHAPAFETAGTYLRLVKINDWTEITQFILEHRARASCKYALPLRIPPAACLTSKAYQQGVTVDSIGMLPRFIPENQRTVALYSLKIAGWHGPKPPYTSTLLPPELSDTTNATQPELVPEDPEDSALLEDPAGTVSSQIPPNWHIPSIQDVAPHHAPAAPSNPGLIIGALAGSTLAVLVIGGIAFWVRRRAQMAPKRLRLPHIRDDDAPPSHQPLFY

| HHV3: Q9J3M8 · GE_VZVO | Envelope glycoprotein E | 623 AA |
| --- | --- | --- |

MGTVNKPVVGVLMGFGIITGTLRITNPVRASVLRYDDFHIDEDKLDTNSVYEPYYHSDHAESSWVNRGESSRKAYDHNSPYIWPRNDYDGFLENAHEHHGVYNQGRGIDSGERLMQPTQMSAQEDLGDDTGIHVIPTLNGDDRHKIVNVDQRQYGDVFKGDLNPKPQGQRLIEVSVEENHPFTLRAPIQRIYGVRYTETWSFLPSLTCTGDAAPAIQHICLKHTTCFQDVVVDVDCAENTKEDQLAEISYRFQGKKEADQPWIVVNTSTLFDELELDPPEIEPGVLKVLRTEKQYLGVYIWNMRGSDGTSTYATFLVTWKGDEKTRNPTPAVTPQPRGAEFHMWNYHSHVFSVGDTFSLAMHLQYKIHEAPFDLLLEWLYVPIDPTCQPMRLYSTCLYHPNAPQCLSHMNSGCTFTSPHLAQRVASTVYQNCEHADNYTAYCLGISHMEPSFGLILHDGGTTLKFVDTPESLSGLYVFVVYFNGHVEAVAYTVVSTVDHFVNAIEERGFPPTAGQPPATTKPKEITPVNPGTSPLLRYAAWTGGLAAVVLLCLVIFLICTAKRMRVKAYRVDKSPYNQSMYYAGLPVDDFEDSESTDTEEEFGNAIGGSHGGSSYTVYIDKTR

| HHV4: P03188 · GB_EBVB9 | Envelope glycoprotein B | 857 AA |
| --- | --- | --- |

MTRRRVLSVVVLLAALACRLGAQTPEQPAPPATTVQPTATRQQTSFPFRVCELSSHGDLFRFSSDIQCPSFGTRENHTEGLLMVFKDNIIPYSFKVRSYTKIVTNILIYNGWYADSVTNRHEEKFSVDSYETDQMDTIYQCYNAVKMTKDGLTRVYVDRDGVNITVNLKPTGGLANGVRRYASQTELYDAPGWLIWTYRTRTTVNCLITDMMAKSNSPFDFFVTTTGQTVEMSPFYDGKNKETFHERADSFHVRTNYKIVDYDNRGTNPQGERRAFLDKGTYTLSWKLENRTAYCPLQHWQTFDSTIATETGKSIHFVTDEGTSSFVTNTTVGIELPDAFKCIEEQVNKTMHEKYEAVQDRYTKGQEAITYFITSGGLLLAWLPLTPRSLATVKNLTELTTPTSSPPSSPSPPAPSAARGSTPAAVLRRRRRDAGNATTPVPPTAPGKSLGTLNNPATVQIQFAYDSLRRQINRMLGDLARAWCLEQKRQNMVLRELTKINPTTVMSSIYGKAVAAKRLGDVISVSQCVPVNQATVTLRKSMRVPGSETMCYSRPLVSFSFINDTKTYEGQLGTDNEIFLTKKMTEVCQATSQYYFQSGNEIHVYNDYHHFKTIELDGIATLQTFISLNTSLIENIDFASLELYSRDEQRASNVFDLEGIFREYNFQAQNIAGLRKDLDNAVSNGRNQFVDGLGELMDSLGSVGQSITNLVSTVGGLFSSLVSGFISFFKNPFGGMLILVLVAGVVILVISLTRRTRQMSQQPVQMLYPGIDELAQQHASGEGPGINPISKTELQAIMLALHEQNQEQKRAAQRAAGPSVASRALQAARDRFPGLRRRRYHDPETAAALLGEAETEF

| HHV5: P06473 · GB_HCMVA | Envelope glycoprotein B | gB | 906 AA |
| --- | --- | --- | --- |

MESRIWCLVVCVNLCIVCLGAAVSSSSTSHATSSTHNGSHTSRTTSAQTRSVYSQHVTSSEAVSHRANETIYNTTLKYGDVVGVNTTKYPYRVCSMAQGTDLIRFERNIICTSMKPINEDLDEGIMVVYKRNIVAHTFKVRVYQKVLTFRRSYAYIYTTYLLGSNTEYVAPPMWEIHHINKFAQCYSSYSRVIGGTVFVAYHRDSYENKTMQLIPDDYSNTHSTRYVTVKDQWHSRGSTWLYRETCNLNCMLTITTARSKYPYHFFATSTGDVVYISPFYNGTNRNASYFGENADKFFIFPNYTIVSDFGRPNAAPETHRLVAFLERADSVISWDIQDEKNVTCQLTFWEASERTIRSEAEDSYHFSSAKMTATFLSKKQEVNMSDSALDCVRDEAINKLQQIFNTSYNQTYEKYGNVSVFETSGGLVVFWQGIKQKSLVELERLANRSSLNITHRTRRSTSDNNTTHLSSMESVHNLVYAQLQFTYDTLRGYINRALAQIAEAWCVDQRRTLEVFKELSKINPSAILSAIYNKPIAARFMGDVLGLASCVTINQTSVKVLRDMNVKESPGRCYSRPVVIFNFANSSYVQYGQLGEDNEILLGNHRTEECQLPSLKIFIAGNSAYEYVDYLFKRMIDLSSISTVDSMIALDIDPLENTDFRVLELYSQKELRSSNVFDLEEIMREFNSYKQRVKYVEDKVVDPLPPYLKGLDDLMSGLGAAGKAVGVAIGAVGGAVASVVEGVATFLKNPFGAFTIILVAIAVVIITYLIYTRQRRLCTQPLQNLFPYLVSADGTTVTSGSTKDTSLQAPPSYEESVYNSGRKGPGPPSSDASTAAPPYTNEQAYQMLLALARLDAEQRAQQNGTDSLDGQTGTQDKGQKPNLLDRLRHRKNGYRHLKDSDEEENV

| HHV6A: P0DOE0 · GQ2_HHV6U | Envelope glycoprotein Q2 | 214 AA |
| --- | --- | --- |

MHFLVVYILIHFHAYRGMAALPLFSTLPKITSCCDSYVVINSSTSVSSLISTCLDGEILFQNEGQKFCRPLTDNRTIVYTMQDQVQKPLSVTWMDFNLVISDYGRDVINNLTKSAMLARKNGPRYLQMENGPRYLQMETRISDLFRHECYQDNYYVLDKKLQMFYPTTHSNELLFYPSEATLPSPWQEPPFSSPWPEPTFPSRWYWLLLNYTNY

| HHV6B: Q9QJ11 · GQ1_HHV6Z | Envelope glycoprotein Q1 | 516 AA |
| --- | --- | --- |

MRPPRRSAPILVCAISMATALSNATVHRDAGTVESTPPPDDEDNYTAKYYDDSIYFNIYDGTNPTPRRRTLPEIISKFSTSEMSRLGGLKAFVPVDYTPTTTLEDIEDLLNYAICDDNSCGCLIETEARXMFGDIIICVPLSAESRGVRNLKSRIMPMGLSQILSSGLGLHFSLLYGAFGSNYNSLAYMERLKPLTAMTAIAFCPMTSKLELRQNYRLEKARXNLIVNIELLKIQNHGGQTIKTLTSFAIVRKDSDGQDWETCTRFASVSIEDILRSKPAANGTCCPPRDVHHDRPTLQSSNSWTRTEYFEPWQDVVDAYVPINDNHCPNDSYVVFQTLQGHEWCSRLNKNDTKNYLSSVLAFKNALYETEELMETIGMRLASQILSLVGQRGTSIRNIDPAIVSALWHSLPEKLTTTNIKYDIASPTHMSPALXTIFIQTGTSKQRFRNAGLLMVNNIFTVQARYSKQNMFEKKIYGYEHLGQALCEGGHVFYNPRDVYFQNIKMAATEPTVVRT

| HHV7: P52353 · GH_HHV7J | Envelope glycoprotein H | 690 AA |
| --- | --- | --- |

MYFYINSLLLIVSINGWKHWNILNSSICVNEKTNQTIIQPGLITFNFHDYNETRVYQIPKCLFGYTFVSNLFDSVNFDESFDQYKHRITRFFNPSTEKAVKIYAQKFQTNIKPVSHTKTITVSFLPLFYEKDVYFANVSEIRKLYYNQYICTLSNGLTDYLFPITERCVMRHYNYLNTVFMLALTPSFFIISVETGMDDVVFIFGNVSRIFFKAPFRKSSFIYRQTVSDDLLLITKKTTIERFYPFLKIDFLDDIWKQNYDISFLIAKFNKLATVYIMEGFCGKPVNKDTFHLMFLFGLTHFLYSTRGDGLLPLLEILNTHQSIITMGRFLEKCFKMTKSHLLYPEMEKLQNFQLVDYSYITSDLTIPISAKLAFLSLADGRIVTVPQNKWKEIENNIETLYEKHKLFTNLTQPERANLFLLSEIGNSLVFQEKIKRKIHVLLASLCNPLEMYFWTHMLDNVMDIETMFSPCATATRKDLTQRVVNNILSYKNLDAYTNKVMNTLSVYRKKRLDMFKSISCVSNEQAAFLTLPNITYTISSKYILAGTSFSVTSTVISTTIIITVVPLNSTCTPTNYKYSVKNIKPIYNISSHDCVFCESLVVEYDDIDGIIQFVYIMDDKQLLKLIDPDTNFIDVNPRTHYLLFLRNGSVFEITALDLKSSQVSIMLVLLYLIIIIIVLFGIYHVFRLF

| HHV8: F5HAK9 · GH_HHV8P | Envelope glycoprotein H | 730 AA |
| --- | --- | --- |

MQGLAFLAALACWRCISLTCGATGALPTTATTITRSATQLINGRTNLSIELEFNGTSFFLNWQNLLNVITEPALTELWTSAEVAEDLRVTLKKRQSLFFPNKTVVISGDGHRYTCEVPTSSQTYNITKGFNYSALPGHLGGFGINARLVLGDIFASKWSLFARDTPEYRVFYPMNVMAVKFSISIGNNESGVALYGVVSEDFVVVTLHNRSKEANETASHLLFGLPDSLPSLKGHATYDELTFARNAKYALVAILPKDSYQTLLTENYTRIFLNMTESTPLEFTRTIQTRIVSIEARRACAAQEAAPDIFLVLFQMLVAHFLVARGIAEHRFVEVDCVCRQYAELYFLRRISRLCMPTFTTVGYNHTTLGAVAATQIARVSATKLASLPRSSQETVLAMVQLGARDGAVPSSILEGIAMVVEHMYTAYTYVYTLGDTERKLMLDIHTVLTDSCPPKDSGVSEKLLRTYLMFTSMCTNIELGEMIARFSKPDSLNIYRAFSPCFLGLRYDLHPAKLRAEAPQSSALTRTAVARGTSGFAELLHALHLDSLNLIPAINCSKITADKIIATVPLPHVTYIISSEALSNAVVYEVSEIFLKSAMFISAIKPDCSGFNFSQIDRHIPIVYNISTPRRGCPLCDSVIMSYDESDGLQSLMYVTNERVQTNLFLDKSPFFDNNNLHIHYLWLRDNGTVVEIRGMYRRRAASALFLILSFIGFSGVIYFLYRLFSILY

| Severe acute respiratory syndrome coronavirus 2 (2019-nCoV) (SARS-CoV-2) P0DTC2 | Spike glycoprotein | 1273 AA |
| --- | --- | --- |

MFVFLVLLPLVSSQCVNLTTRTQLPPAYTNSFTRGVYYPDKVFRSSVLHSTQDLFLPFFSNVTWFHAIHVSGTNGTKRFDNPVLPFNDGVYFASTEKSNIIRGWIFGTTLDSKTQSLLIVNNATNVVIKVCEFQFCNDPFLGVYYHKNNKSWMESEFRVYSSANNCTFEYVSQPFLMDLEGKQGNFKNLREFVFKNIDGYFKIYSKHTPINLVRDLPQGFSALEPLVDLPIGINITRFQTLLALHRSYLTPGDSSSGWTAGAAAYYVGYLQPRTFLLKYNENGTITDAVDCALDPLSETKCTLKSFTVEKGIYQTSNFRVQPTESIVRFPNITNLCPFGEVFNATRFASVYAWNRKRISNCVADYSVLYNSASFSTFKCYGVSPTKLNDLCFTNVYADSFVIRGDEVRQIAPGQTGKIADYNYKLPDDFTGCVIAWNSNNLDSKVGGNYNYLYRLFRKSNLKPFERDISTEIYQAGSTPCNGVEGFNCYFPLQSYGFQPTNGVGYQPYRVVVLSFELLHAPATVCGPKKSTNLVKNKCVNFNFNGLTGTGVLTESNKKFLPFQQFGRDIADTTDAVRDPQTLEILDITPCSFGGVSVITPGTNTSNQVAVLYQDVNCTEVPVAIHADQLTPTWRVYSTGSNVFQTRAGCLIGAEHVNNSYECDIPIGAGICASYQTQTNSPRRARSVASQSIIAYTMSLGAENSVAYSNNSIAIPTNFTISVTTEILPVSMTKTSVDCTMYICGDSTECSNLLLQYGSFCTQLNRALTGIAVEQDKNTQEVFAQVKQIYKTPPIKDFGGFNFSQILPDPSKPSKRSFIEDLLFNKVTLADAGFIKQYGDCLGDIAARDLICAQKFNGLTVLPPLLTDEMIAQYTSALLAGTITSGWTFGAGAALQIPFAMQMAYRFNGIGVTQNVLYENQKLIANQFNSAIGKIQDSLSSTASALGKLQDVVNQNAQALNTLVKQLSSNFGAISSVLNDILSRLDKVEAEVQIDRLITGRLQSLQTYVTQQLIRAAEIRASANLAATKMSECVLGQSKRVDFCGKGYHLMSFPQSAPHGVVFLHVTYVPAQEKNFTTAPAICHDGKAHFPREGVFVSNGTHWFVTQRNFYEPQIITTDNTFVSGNCDVVIGIVNNTVYDPLQPELDSFKEELDKYFKNHTSPDVDLGDISGINASVVNIQKEIDRLNEVAKNLNESLIDLQELGKYEQYIKWPWYIWLGFIAGLIAIVMVTIMLCCMTSCCSCLKGCCSCGSCCKFDEDDSEPVLKGVKLHYT

| P0CL66 · OSPA_BORBU | Outer surface protein A (Borrelia burgdorferi) | 273 AA |
| --- | --- | --- |

MKKYLLGIGLILALIACKQNVSSLDEKNSVSVDLPGEMKVLVSKEKNKDGKYDLIATVDKLELKGTSDKNNGSGVLEGVKADKSKVKLTISDDLGQTTLEVFKEDGKTLVSKKVTSKDKSSTEEKFNEKGEVSEKIITRADGTRLEYTGIKSDGSGKAKEVLKGYVLEGTLTAEKTTLVVKEGTVTLSKNISKSGEVSVELNDTDSSAATKKTAAWNSGTSTLTITVNSKKTKDLVFTKENTITVQQYDSNGTKLEGSAVEITKLDEIKNALK

| Q07337 · OSPC_BORBU | Outer surface protein C (Borrelia burgdorferi) | 210 AA |
| --- | --- | --- |

MKKNTLSAILMTLFLFISCNNSGKDGNTSANSADESVKGPNLTEISKKITDSNAVLLAVKEVEALLSSIDEIAAKAIGKKIHQNNGLDTENNHNGSLLAGAYAISTLIKQKLDGLKNEGLKEKIDAAKKCSETFTNKLKEKHTDLGKEGVTDADAKEAILKTNGTKTKGAEELGKLFESVEVLSKAAKEMLANSVKELTSPVVAESPKKP

| O50917 · DPBA_BORBU | Decorin-binding protein A (Borrelia burgdorferi) | 191 AA |
| --- | --- | --- |

MIKCNNKTFNNLLKLTILVNLLISCGLTGATKIRLERSAKDITDEIDAIKKDAALKGVNFDAFKDKKTGSGVSENPFILEAKVRATTVAEKFVIAIEEEATKLKETGSSGEFSAMYDLMFEVSKPLQKLGIQEMTKTVSDAAEENPPTTAQGVLEIAKKMREKLQRVHTKNYCTLKKKENSTFTDEKCKNN

| Q6RH12 · OppA-2_BORBG | OppA-2 (Borrelia burgdorferi) | 107 AA |
| --- | --- | --- |

RAGWIGDYADPLTFLSIFTQGYTQFSSHNYSNPEYNELIKKSDLELDPIKRQDILRQAEEIIIEKDFPIAPIYIYGNSYLFRNDKWTGWNTNFLERFDLCQLKLKNK

| O06878 · Vlse_BORBG | Variable large protein (Borrelia burgdorferi | 356 AA |
| --- | --- | --- |

MKKISSAILLTTFFVFINCKSQVADKDDPTNKFYQSVIQLGNGFLDVFTSFGGLVAEAFGFKSDPKKSDVKTYFTTVAAKLEKTKTDLNSLPKEKSDISSTTGKPDSTGSVGTAVEGAIKEVSELLDKLVKAVKTAEGASSGTAAIGEVVADADAAKVADKASVKGIAKGIKEIVEAAGGSEKLKAVAAAKGENNKGAGKLFGKAGAAAHGDSEAASKAAGAVSAVSGEQILSAIVTAADAAEQDGKKPEEAKNPIAAAIGDKDGGAEFGQDEMKKDDQIAAAIALRGMAKDGKFAVKDGEKEKAEGAIKGAAESAVRKVLGAITGLIGDAVSSGLRKVGDSVKAASKETPPALNK

**Table S2**. Amino acid sequences of PBBA epitopes including their location (Start, End) in the AA sequence of the corresponding protein (Table S1). Epitope length is 9-mer for HLA Class I alleles (B*08:01, C*07:04) and 15-mer for Class II alleles (DPB1:02:01, DQB1*03:03). Blue and red indicate protective and risk alleles, respectively.

| Pathogen | Allele | Protein | Start | End | Peptide sequence | PBBA (nM) |
| --- | --- | --- | --- | --- | --- | --- |
| HHV1 | HLA-B*08:01 | Q69091 | 363 | 371 | WMRRHTQKA | 32.1 |
| HHV2 | HLA-B*08:01 | P03172 | 112 | 120 | EARKHTYNL | 45.5 |
| HHV3 | HLA-B*08:01 | Q9J3M8 | 563 | 571 | RMRVKAYRV | 81.8 |
| HHV4 | HLA-B*08:01 | P03188 | 1 | 9 | MTRRRVLSV | 9.2 |
| HHV5 | HLA-B*08:01 | P06473 | 630 | 638 | YLFKRMIDL | 30.3 |
| HHV6A | HLA-B*08:01 | P0DOE0 | 200 | 208 | FPSRWYWLL | 163.8 |
| HHV6B | HLA-B*08:01 | Q9QJ11 | 20 | 28 | NLKSRIMPM | 4.8 |
| HHV7 | HLA-B*08:01 | P52353 | 264 | 272 | FLIAKFNKL | 24.2 |
| HHV8 | HLA-B*08:01 | F5HAK9 | 346 | 354 | YFLRRISRL | 33.8 |
| HHV1 | HLA-C*07:04 | Q69091 | 125 | 133 | FRMGGNCAI | 747.6 |
| HHV2 | HLA-C*07:04 | P03172 | 125 | 133 | YRMGDNCAI | 1432.7 |
| HHV3 | HLA-C*07:04 | Q9J3M8 | 481 | 489 | YFNGHVEAV | 1155.4 |
| HHV4 | HLA-C*07:04 | P03188 | 276 | 284 | FLDKGTYTL | 664.6 |
| HHV5 | HLA-C*07:04 | P06473 | 153 | 161 | YAYIYTTYL | 1000.1 |
| HHV6A | HLA-C*07:04 | P0DOE0 | 15 | 23 | YRGMAALPL | 1753.1 |
| HHV6B | HLA-C*07:04 | Q9QJ11 | 13 | 21 | FRNAGLLMV | 1421.1 |
| HHV7 | HLA-C*07:04 | P52353 | 145 | 153 | YYNQYICTL | 897.0 |
| HHV8 | HLA-C*07:04 | F5HAK9 | 496 | 504 | YRAFSPCFL | 570.5 |
| HHV1 | DPB1*02:01 | Q69091 | 181 | 195 | PAFETAGTYLRLVKI | 125.8 |
| HHV2 | DPB1*02:01 | P03172 | 342 | 356 | IIGALAGSTLAVLVI | 117.8 |
| HHV3 | DPB1*02:01 | Q9J3M8 | 188 | 202 | IQRIYGVRYTETWSF | 42.3 |
| HHV4 | DPB1*02:01 | P03188 | 553 | 567 | SRPLVSFSFINDTKT | 59.3 |
| HHV5 | DPB1*02:01 | P06473 | 135 | 149 | AHTFKVRVYQKVLTF | 36.3 |
| HHV6A | DPB1*02:01 | P0DOE0 | 87 | 101 | KPLSVTWMDFNLVIS | 45.0 |
| HHV6B | DPB1*02:01 | Q9QJ11 | 178 | 192 | PAIVSALWHSLPEKL | 22.5 |
| HHV7 | DPB1*02:01 | P52353 | 259 | 273 | NYDISFLIAKFNKLA | 5.6 |
| HHV8 | DPB1*02:01 | F5HAK9 | 50 | 64 | ELEFNGTSFFLNWQN | 14.3 |
| HHV1 | DQB1*03:03 | Q69091 | 172 | 186 | DNLGFLMHAPAFETA | 1275.7 |
| HHV2 | DQB1*03:03 | P03172 | 172 | 186 | DNLGFLMHAPAFETA | 1275.7 |
| HHV3 | DQB1*03:03 | Q9J3M8 | 536 | 550 | LRYAAWTGGLAAVVL | 1411.2 |
| HHV4 | DQB1*03:03 | P03188 | 416 | 430 | SAARGSTPAAVLRRR | 1431.9 |
| HHV5 | DQB1*03:03 | P06473 | 490 | 504 | LRGYINRALAQIAEA | 872.9 |
| HHV6A | DQB1*03:03 | P0DOE0 | 12 | 26 | FHAYRGMAALPLFST | 1111.7 |
| HHV6B | DQB1*03:03 | Q9QJ11 | 10 | 24 | KQRFRNAGLLMVNNI | 1335.5 |
| HHV7 | DQB1*03:03 | P52353 | 267 | 281 | AKFNKLATVYIMEGF | 1712.9 |
| HHV8 | DQB1*03:03 | F5HAK9 | 366 | 380 | HTTLGAVAATQIARV | 1482.7 |
| *B. burgdorferi* | HLA-B*08:01 | O50917 | 80 | 88 | EAKVRATTV | 68.3 |
| *B. burgdorferi* | HLA-C*07:04 | P0CL66 | 237 | 245 | FTKENTITV | 1410.7 |
| *B. burgdorferi* | DPB1*02:01 | Q07337 | 170 | 184 | AEELGKLFESVEVLS | 71.4 |
| *B. burgdorferi* | DQB1*03:03 | O06878 | 229 | 243 | EQILSAIVTAADAAE | 1028.2 |
| SARS-CoV-2 | HLA-B*08:01 | P0DTC2 | 269 | 277 | YLQPRTFLL | 19.9 |
| SARS-CoV-2 | HLA-C*07:04 | P0DTC2 | 269 | 277 | YLQPRTFLL | 362.3 |
| SARS-CoV-2 | DPB1*02:01 | P0DTC2 | 340 | 354 | EVFNATRFASVYAWN | 5.4 |
| SARS-CoV-2 | DQB1*03:03 | P0DTC2 | 1010 | 1024 | QQLIRAAEIRASANL | 614.1 |
